# Clarifying the relation between mechanistic explanations and reductionism

**DOI:** 10.3389/fpsyg.2023.984949

**Published:** 2023-04-25

**Authors:** Mark Couch

**Affiliations:** Department of Philosophy, Seton Hall University, South Orange, NJ, United States

**Keywords:** mechanistic explanation, reduction, wholes, neuroscience, action potential

## Abstract

The topic of mechanistic explanation in neuroscience has been a subject of recent discussion. There is a lot of interest in understanding what these explanations involve. Furthermore, there is disagreement about whether neurological mechanisms themselves should be viewed as reductionist in nature. In this paper I will explain how these two issues are related. I will, first, describe how mechanisms support a form of antireductionism. This is because the mechanisms that exist should be seen as involving part-whole relations, where the behavior of a whole is more than the sum of its parts. After this, I will consider mechanistic explanations and how they can be understood. While some people think the explanations concern existing entities in the world, I will argue that we can understand the explanations by viewing them in terms of arguments. Despite the fact that it is possible to understand mechanistic explanations in this manner, the antireductionist point remains.

## 1. Introduction

The topic of mechanistic explanations has been an issue of recent interest among philosophers and scientists. It is evident to many researchers that an appeal to mechanisms plays an important role in the sciences. For instance, neuroscientists have explained the signaling by the action potential in the neuron in terms of the physical mechanism that underlies this phenomenon. The action potential is taken to be a result of the components and their behaviors in the neuron that give rise to this distinctive capacity. Furthermore, we can understand the action potential not in terms of any individual component, but as in some way a product of a set of components working together in an organized manner. In this way the phenomenon can be viewed as a higher-level behavior of a neurological mechanism that is not reducible to its lower-level components. While the behavior of the mechanism cannot be reduced to the individual components, it is still dependent upon them.

Just how to think of mechanisms like this and how they should be understood is the topic of this paper. My aim will be to describe how we should think about mechanistic explanations and how this relates to reductionism. After introducing the subject in this first part, I will go on in section 2 to describe how I think we should understand mechanisms. I will offer an account of mechanisms that explains the features they have, including the idea that mechanisms should be viewed as wholes that are made from a collection of parts. In section 3, I will explain why this is a nonreductive way of thinking about mechanisms when considered in terms of how mechanisms exist in the world. In section 4, I will develop this by discussing a variety of reasons for why mechanisms are nonreductive. After this in section 5, I will turn from mechanisms as they exist to the notion of mechanistic explanation and describe how this too should be understood. In my view the notion of explanation should be understood as including both representational features and ontological features that are needed for characterizing mechanisms. I will explain why this view is in contrast to other views that are more ontologically focused. Furthermore, I will offer a view of the explanations which takes them to be expressible in terms of arguments that consist of statements. While this view is not as common as it once was I think it can still be useful. In section 6, I will describe the implications of this way of thinking about mechanistic explanation for the notion of reductionism, and suggest that the antireductionist view of mechanisms described before is consistent with this perspective. In the last section 7 I will draw some conclusions for how these two issues are related.

## 2. Understanding mechanisms

It will help to begin with an account of mechanisms and how they should be understood. Here I am talking about mechanisms themselves and, as we might say, how they exist in nature.

While the notion of a mechanism is commonly appealed to in the sciences it is not entirely clear how this notion should be analyzed. There are different ways that people have offered for thinking about this. These different accounts sometimes emphasize different features, or include subtle differences to note about what makes something a mechanism. Since this is not the place to review these discussions in detail what I will do is begin with an account that I think captures the main features that need to be included. This is a way of thinking about mechanisms that has been presented by Bechtel and Abrahamsen (2005) and is often appealed to by others. As they write, “A mechanism is a structure performing a function in virtue of its component parts, component operations, and their organization. The orchestrated functioning of the mechanism is responsible for one or more phenomena” (423). To understand this characterization of a mechanism, we will need to explain each of the notions it mentions.

We can begin with the notion of a “function,” which can be understood in different ways that need to be distinguished from each other. I have said that a mechanism is a structure that consists of components working together to produce a behavior of the mechanism. This behavior is what I mean by the functioning of the mechanism. The notion of functioning at work here refers to what is sometimes called the “causal role” exhibited by the mechanism (Cummins, 1975), or what I will refer to as its “behavior” (Glennan, 2017, p. 24). For instance, because the behavior of the nerve cell is to transmit electrical signals through the neural system we say that “it functions to transmit electrical signals.” Notice that with this approach to the notion we are not including any biological purposes or goals that are due to the evolutionary history of a mechanism. The evolutionary notion of function is important in those disciplines concerned with why a trait evolved due to some kind of selective pressures, but this sort of notion is distinct from the one we are concerned with. We can describe the behavior of the nerve cell in the neural system independently from saying anything about its evolutionary history.

One thing to note is that there’s an ambiguity that occurs in how we talk about behaviors, since we sometimes talk about the performance of a behavior, or the capacity to perform a behavior. The performance of a behavior involves its actual occurrence, while a capacity concerns the presence of an ability that can be manifested. We say, for instance, that the nerve cell has the capacity to produce a signal in the nervous system even in its resting state. I will follow Cummins in allowing that both kinds of notions should be included in our account.^1^

After this the next notion to consider concerns the “components” of a mechanism and their behaviors; these can be understood to be the parts of a mechanism that contribute to its operation. A mechanism will typically contain a number of parts but not all of these will serve as working components. An example of this would be chemicals introduced into a neuron that do not affect its operation, which are in a sense “a part” contained within the brain. But these are not working parts whose behaviors help them to contribute to a behavior of the mechanism. The notion of a component refers to those parts within the mechanism that contribute to its behavior, not those parts which are merely present in the system in some way. The nerve cells in the brain that contribute to the signaling system will count as components in the system by this criterion.

The last notion to consider in characterizing a mechanism is the “organization” of the components. A mechanism is not merely a set of components taken by themselves, but concerns a set of components that have been organized somehow to produce a behavior. The components of a nerve cell do not produce the cell’s overall behavior individually, but work together in an organized manner to produce the signals. This organization of the components should be understood to include things like the causal, spatial, and temporal organization they exhibit. For instance, the physical events that constitute the action potential in the nerve cell concern the signal, which involves a sequence of steps, beginning with the opening of channels, an influx of ions across the cell membrane, a change in the resting potential, and then a response signal. Each of these events occurs in order and explaining the behavior of the cell requires describing how the components present behave in an organized way. So this is an important feature of the components to include in describing the features of the mechanism.

Understood this way, a mechanism should be viewed as a set of components whose coordinated behavior results in the behavior of the system as a whole. In this sense, a mechanism consists of a collection of interacting parts that underlies a particular behavior. This notion of a mechanism appears to play a central role in fields like neuroscience, which is concerned with investigating the systems of the brain and how they underlie our mental capacities. An understanding of our mental capacities leads researchers to be interested in the details of the mechanisms and how they should be understood. Given their role, it is important to be clear about the features of mechanisms and what their study can teach us about this area of the sciences.

## 3. Antireductionism in mechanisms

Up to this point, I have described the behavior of a mechanism as a whole in relation to the behavior of its components. I need to say something more about this issue, since our concern is with trying to understand what the study of mechanisms tells us about reductionism in the area of the sciences we are concerned with. Let us begin with the idea that mechanisms as a whole are constituted by the components that make them up. The components are the individual parts that contribute to a behavior of a mechanism. The mechanism as a whole can be understood as the group of parts-plus-their-organization that serves as the behaving unit. They are related to one another in the sense that there is a form of part-whole relation between the components and the mechanism as a whole.^2^ This point needs to be described carefully to make sense of the features of mechanisms with which we are concerned.

This way of describing mechanisms has been characterized by Craver (2007, p. 188) in terms of the notion of “levels of mechanisms.” The idea is that the components of a mechanism should be seen as at a lower level than the mechanism as a whole, and that organizing the components together results in a higher level of mechanism.^3^ For instance, the intracellular components in the nerve cell that were described are the lower-level components that serve to make up the higher-level mechanism of the cell as a whole. In this approach to mechanisms, the intracellular components should be understood as individual entities with their behaviors. These entities and their behaviors constitute the mechanism as a whole, which consists in another individual with its behaviors. So in this approach the mechanism should be understood to involve a relation between different individuals that exist (an individual is an entity that is capable of independent existence). It should also be mentioned that there is another notion that is sometimes appealed to in this area by philosophers that concerns a relation between the properties of an object. This notion is called “levels of realization” (Craver, 2007, p. 165) and refers to a different notion. This notion is different from the one we are concerned with about individuals since it concerns relations between properties. As Craver suggests, the right way to think about the mechanisms we are considering is to view them as complex systems constituted by individual components that work together to produce a behavior of the whole.

On this way of thinking it follows that mechanisms as a whole have behaviors that their individual components lack; we can see how this works in terms of the example being used. The nerve cell has the behavior of sending an electrochemical signal to other neural cells in the brain, which is a behavior of the whole cell. But this behavior of the cell is not a behavior of any of its intracellular components individually. The ions that flow into the channels of the cell do not themselves have the behavior of sending electrochemical signals through the axon; they are merely one component that (partially) contributes to this behavior. It is also important to see that the behavior of the cell is not a result of adding the contributions of the ions and other parts together in a simple way. In some structures, when we add the components together the result is a property that differs from the components. An illustration would be the weight of a pile of sand that simply results from adding together the weights of the individual grains. But the behavior one finds in the nerve cell is not like this since it depends on the different interrelations among the components that include the channels, ions, and changing resting potential. The channels have to open and allow the ions to enter, which produces a change in the resting potential, and as this changes new channels open and close to facilitate the signal through the axon. It is not a simple relationship that is involved like with the grains of sand but a situation where the behavior that results from the organization of the complex is more than the sum of its parts (Craver, 2007, p. 216; *cf.* Biem Graben, 2016).^4^ In this respect, the cell as a whole should be seen to have behaviors that are distinct and novel from the behaviors of its components. It is this aspect of the nerve cell that distinguishes it from other kinds of cases that is characteristic of the mechanisms we are examining.

The behaviors of a whole are important to recognize for understanding mechanisms. This is because they enable the mechanisms to make new kinds of causal contributions. As Craver puts it, “wholes have causal powers that their parts individually do not have” (Craver, 2007, p. 214). There are causal powers at the level of the whole mechanism that are distinct from the causal powers at the level of the components. In terms of our example, due to its organization the causal powers of the nerve cell as a whole are distinct from the causal powers of the components that make it up. The cell as a whole causally contributes to the transmission of information through the signaling system. But the ions in the cell do not directly do this. In this respect, the causal powers of the entities are different because the causal relationships in which they participate are different. Because of this the mechanism is capable of entering into different interactions and so causally contributes something new to the world aside from the components.

One thing to add is that, in saying the behavior of the whole mechanism is more than the sum of its parts, I am not intending to deny that mechanisms are constituted by the physical entities and behaviors that make them up. There is a notion of antireductionism according to which the higher-level behaviors of a system go beyond the organized interactions of the parts [*cf.* strong emergentism (Craver, 2007, p. 216)], but that is not what I am claiming. The idea is that there are complex interactions among the components of the system that produce a new behavior of the whole, but where this whole is constituted out of parts and their behaviors that make it up. So the form of antireductionism being described is consistent with the idea that the resulting behavior is dependent on the components. What matters to the account offered is the idea that various lower-level entities in the world can become organized together in certain ways, giving rise to new properties and behaviors at higher levels. These higher-level mechanisms are made out of lower-level constituents, but they cannot be reduced to the constituents.

## 4. Versions of reductionism

To clarify this point it will help to consider some notions of reductionism and explain whether any of these notions apply to the notion of mechanisms I have described. Here I will consider three common ways of thinking about ontological reductionism one hears.

On one of the common ways of thinking about reductionism over the years, this concerns a relation between different types of properties. The idea is that we have a reduction when a higher-level property of an entity is shown to be the same as some lower-level property of the entity (Sklar, 1967; Kim, 1998). On this view a property can be reduced to another property just in case the former is type identical with the latter. For instance, if we can identify the higher-level property of “being water” with the lower-level property of “being H_2_O,” then we have shown that being water can be reduced to being H_2_O and there is really no difference between these properties.

The view I have presented of mechanisms is inconsistent with this point. The account offers a way of thinking about the relation between higher and lower-level mechanisms in which they involve distinct individuals and properties. It was noted before, for instance, that the behavior of wholes involves distinct individuals from the behaviors of the components. What is going on in the neural cell as a whole when it signals is distinct from what is going on with the individual ions. The lower-level behaviors of the components contribute to but are distinct from the higher-level behavior of the cell. This point has been explained by Gillett in terms of the “qualitative distinctness” of the properties of different individuals. In his account of mechanisms there are distinct levels of individuals and these come with corresponding distinct levels of behaviors (Gillett, 2010, 2022). Because there are qualitatively distinct behaviors like this that are not shared we should not think of the behaviors of the whole as just a subset (or part) of the behaviors of the components (for an alternative account see Piccinini (2022a,b)). As a result of all this, there is no identification to make among the higher and lower-level properties and so no reduction which exists. This point can also be combined with the point that there are sometimes different lower-level mechanism types which can underlie the same type of higher-level mechanism in the sense of multiple realization (*cf.* Piccinini, 2020). For instance, it is possible that the type “neural signal” can be produced in different ways in different neurons, say with different numbers of ions and channels that have different spatial organization throughout the cell. In this respect there is no identification to make between the types present.

A second notion of reduction involves the idea that lower levels of mechanisms explain higher-level behaviors without intermediate explanatory levels, in the sense that the lower levels directly account for the higher levels. These lower-level components are what matter fundamentally and scientists should focus their attention on these in their research. An approach like this is represented by ruthlessly reductionist views of neuroscience (Bickle, 2003, 2020) that hold that lower levels of mechanisms are what matter for how mechanisms work.

The view I presented of mechanisms does not fit with this either. The account offers a way of thinking about the notion of mechanisms in which they consist of wholes that are different from the components. Due to their organization higher-level mechanisms can do things that cannot be accounted for in terms of the behavior of lower-level components alone and need to be studied in their own terms. The neural signal, for instance, results from the components operating together at the level of the whole and this needs to be cited for a full explanation. This point has been made by Bechtel who notes that “typically the behavior of the whole system must be studied at its own level with appropriate tools for that level. Research at the level of whole systems … studies, using its own modes of investigation, phenomena different from those studied at the level of the component parts” (Bechtel, 2008, p. 129). Think, for instance, of how researchers might electrically stimulate a whole neural cell to see how it behaves in response. Accounting for the behavior of this sort of case will be done in terms of interventions upon the mechanism as a whole and is not a strictly lower-level affair about individual components. The lower-level components have a contribution to make, but this does not replace the contribution of the whole mechanism.

The third notion I will consider is that mechanisms are reducible in the sense that there is a decomposition of a mechanism’s behavior into the components and their behaviors, so that there is a one-to-one mapping that is preserved. This notion of strong decomposition may also include the idea that in a mechanism individual components and their behaviors can be studied separately from other components in the mechanism (Kaiser and Krickel, 2017).

The problem with this way of thinking about reductionism is that there are often facts about the interrelations of components in a mechanism that affect the behaviors of the components that occur. What happens in a neural cell is not a simple sequence of steps within the cell but a complex set of interacting components behaving together. For instance, the channels in the cell membrane behave by both opening and closing, and this occurs at different rates, and which behavior is performed depends on what the different concentrations of ions are elsewhere in the cell. As a result these other components affect the behavior of the channels and their properties. To know why a channel behaves the way it does one thus has to know about what else is going on in the cell. Because of this the strong notion of decomposition does not seem to apply in this sort of case (Andersen, 2014; Burnston, 2021; Silberstein, 2021). Accepting this is not to deny that a mechanism’s behavior can be explained more weakly in some sense in terms of the behavior of the components and their affects on each other. But this notion of decomposition does not require the stronger notion which is sometimes associated with reductive ways of thinking about mechanistic explanations.

There is more to say about the notion of reductionism than I have said so far and I am not suggesting that what I’ve said on this is complete. What I have been trying to do is to describe how to think about neural mechanisms and their behaviors in a way that I think can be supported by the examples. It seems to me that when we consider the mechanisms that exist, they are best described as involving new behaviors from their components and require study in their own terms, and in this sense we cannot reduce the mechanisms to their components’ properties and behaviors. It should be allowed that there may be other notions of reduction that have different implications in this area since there are different notions that have been offered by people.^5^ Some of the concerns with other ways of thinking about reductionism will be considered at a later point.

## 5. The explanation of mechanisms

So far I have been describing how I think we should view mechanisms as they exist in the world. The account has been concerned with the features of mechanisms, and the entities and behaviors that make them up. I think it is helpful to be concerned with this aspect of mechanisms because we want an adequate account of mechanisms as they exist. What I want to do at this point, though, is turn from questions about how to understand mechanisms to questions about how to explain them. To do this, I will need to say something about the notion of explanation and how it should be understood in this context.

At a general level, when we are concerned with the explanation of a mechanism, we are concerned with providing the reasons why something has occurred in the mechanism. The explanation involves accounting for why that something has occurred. When we apply this sort of idea to explaining the behavior of a mechanism, this means the behavior will be explained in terms of the features that bring it about. We have seen that this consists in referring to the components and their behaviors and how they are organized to produce the behavior. In this sense, it is the reference to the details of the components and their organization that provide the explanation.

This way of describing the explanation comes from a way of thinking about how they should be characterized that’s become widely accepted more recently (Craver, 2007). In the account Craver presents, he is interested in describing the notion of explanation and how it applies to mechanisms in connection to earlier work from Salmon (1984). In the approach Craver takes, an explanation occurs when we have exhibited the entities in the world that serve to bring the phenomenon about. The world consists of entities that stand in causal and other relations to one another, in a temporal and spatial framework. To explain a phenomenon in this framework is to situate it in this causal structure. For instance, think of how we might explain the presence of water on the street after it rains. The explanation would consist of referring to the factors in the environment that served to bring the rain about, which include things like the condensation in the atmosphere and the effects of gravity. We have explained why the street is wet when we have exhibited the factors in the world whose presence led to this phenomenon occurring.

This way of talking about explanation sometimes leads Craver to say that an explanation concerns objective features of the world. To explain why something occurs we have to describe how it fits within the objective structure that exists. But Craver does not limit himself to these objective aspects in talking about the notion of explanation, since he sometimes seems to allow that there is also a role for representations to play. This is because in giving an explanation humans make use of representations of different kinds. This can be understood to mean that explanations involve the use of representations (or conceptual vehicles) that are part of the explanation being offered by someone. In the example of explaining why the street is wet, for example, we have to characterize the phenomenon in terms of the representations “gravity” and “atmospheric condensation,” and describe how these are related to each other to produce the “rain.” This seems to be a common feature of giving explanations since we exchange information with others by means of representations. To include this other aspect in the account we should accept that the activity of giving explanations involves reference to features of the world and includes a means for representing them in language or other forms of representation. In this way of viewing the notion of explanation I described it has both objective and representational aspects [for discussion of this approach see Illari (2013)]. Though it has not always been clear in his account, I think this sort of approach is consistent with what Craver says since he makes reference in his work to “explanatory texts” in places (Craver, 2007, p. 27) that he takes to be representational. While he tends to emphasize the world having objective structure, there is more to explanation than this. I will follow him in including these representational aspects since it is helpful to view explanation as involving both of these together.

Having said this about the explanation of mechanisms, there is a further issue to be addressed. Something needs to be said about the kinds of representations that one might use. There are different types of representations which one may want to make use of in an explanation, which include linguistic, visual, and other forms of representation. The approach I will take on this departs from Craver and comes from an earlier way of thinking about explanation associated with Hempel which characterizes them in terms of a type of argument (Hempel and Oppenheim, 1948). The idea is that we can characterize the explanatory factors of a mechanism in terms of the premises of an argument, from which a conclusion describing the phenomenon to be explained can be derived. The premises will consist of sentences describing the features of the mechanism, and the conclusion will consist of a sentence describing the phenomenon at issue. The explanation will then consist in showing how the conclusion concerning the phenomenon follows from the information contained in the premises. This way of viewing an explanation descends from earlier work which has been influential. But we need to be careful here since not everyone agrees with the idea that explanations should be understood as arguments made of sentences. My approach to this issue will be to follow Hausman (1998) in thinking that explanations can at least be represented in this way, and that there is something helpful in doing this.^6^ This is because it will show how this common form of representation can be used. Furthermore, it is not always clear to everyone what such an approach would look like and it may help to see this laid out carefully. In saying this I am not taking myself to have settled whether this is the only way of thinking about the notion of explanation one might accept. Discussing this would require more time than I can devote to this issue in this setting and a full account will have to be left for another occasion. What I will do is merely show that there is a way of describing mechanistic explanations in this manner that is plausible and illustrate the form such an approach might take.

It will help to provide a more specific example of what a mechanistic explanation will look like along these lines. The basic idea will involve explaining why a mechanism O has a behavior. The explanation will involve analyzing O in terms of the behavior of its components and their organization in the mechanism that enables it to perform the behavior, and representing this in terms of an argument (*cf.* Levine, 2001, p. 74). Here is what this might look like with the example that we have been using. Suppose we are interested in explaining why an action potential is propagated down the axon in a particular cell. We can say that the behavior to be explained is the behavior for having an action potential. The first step in the explanation is to characterize the properties that define the behavior, which consist of the precipitating and manifestation conditions for the behavior. In the example that we are discussing, being an action potential is a behavior of a structure that results from inputs to some components and their behaviors that leads to signals being propagated down a cell. Once we have specified the behavior in this way, the next step is to describe the particular components and their behaviors in a mechanism that lead to this behavior. This is done by identifying the components and behaviors in the mechanism and how they are organized to result in the behavior. Once this is done we have explained why the behavior occurs.

We can lay out the steps of such an explanation in the following way:

1. Having an action potential = df having some components and behaviors caused by inputs to a cell, and that leads to a signal down the axon.
2. The presence of input states causes components and behaviors in organization S, and this leads to a signal down the axon.
3. Mechanism O has components and behaviors in organization S.
4. Thus, mechanism O has the behavior of an action potential.

In the nervous system, the components and behaviors in organization S will consist of the opening of channels, an influx of ions across the membrane, an increase in resting potential, and the initial signal. When these are present they lead to the propagation of the electrical signal down the cell.^7^ The explanation that is offered consists of an argument whose conclusion is that the mechanism has the behavior for an action potential. The explanation is such that the information described in the premises leads to the conclusion regarding the presence of the behavior that is at issue. The explanation works by describing the sequence of events in the mechanism and their order that result in the behavior.

Note that this way of characterizing an explanation is different from Hempel’s earlier account of explanation in certain ways. In particular, notice that there is no requirement that the premises of the explanation include a law of nature, as Hempel required. The first line of the explanation in the account is not a law of nature in the traditional sense, but merely serves to specify a behavior that a mechanism can have. So the account is different from Hempel’s Deductive-Nomological approach that was concerned with explanation in terms of laws. One of the reasons for this is that Hempel was interested in causal explanations between events, which are different from the examples I am considering. The examples I am considering are concerned with explaining how a mechanism underlies a behavior or capacity. With this form of explanation it is not important to describe laws of nature which may (or may not) apply to the mechanism and how they are involved. The explanation is merely concerned with referring to the features within the mechanism whose occurrence underlie the behavior at issue. Representing this information in the explanation enables us to see why the behavior follows from the features described. In doing this, the explanation makes use of arguments to present this information, but in other respects it is different from Hempel’s account.

A further feature of the account to note is that it is consistent with the earlier point that there are both objective and representational aspects to the explanation. On the one hand, there is the mechanism with its features in the world which exists independently from us. The behavior of the mechanism occurs in the world and depends on the other features that make up the mechanism. On the other hand, the explanation is presented in the form of an argument that conveys the information about how the different features of the mechanism are related. By describing the components and how they are organized to bring about the mechanism’s behavior in the premises, we can make sense of why the behavior occurs. This way of thinking about the explanation is useful because the form of argument makes clear the sequence of changes the mechanism undergoes that enables us to understand why the behavior occurs. Furthermore, it should be apparent that the explanation is distinct from the mechanism and merely provides a means for representing information about the mechanism. In this respect, the account is different from Hempel’s approach since he appeared to think that the causal relations some thought existed in the world could be captured entirely in terms of the explanatory information presented in an explanation. This is not a feature of the account I have offered. The account holds that there is a difference between the mechanism in the world and the information in the explanation which serves to represent it.

I think this approach can provide us with a way of understanding the explanation of mechanisms that is useful for thinking about how the explanations work. It allows us to describe the explanation in terms of a common form of representation, and makes clear the different features that are involved in the explanation. There are other aspects of the notion of explanation that one may want to consider in thinking about this notion and I have not tried to address all the concerns that may exist.^8^ Rather than take up all of these issues which need separate treatment, what I want to do is consider how the approach relates to the previous account of mechanisms offered. If the account of mechanistic explanations that was presented can be made to work, what implications does this have with respect to the issue of reductionism?

## 6. Some implications

Let me return to the issue of reductionism in relation to these concerns. There are several implications that would appear to follow from the approach that was offered for this issue.

The first point to observe has to do with the character of the explanations given. We have seen that it was a feature of the approach that an explanation consists in the information in the premises leading to the information in the conclusion listed. The idea is that we have explained a mechanism’s behavior when we have shown how a description of it follows from the information about the mechanism’s components, behaviors, and organization. In this respect, the information about the mechanism’s behavior can be derived from information about the different features of the mechanism that are referenced. Given this, one might think that the account is in tension with the earlier point that the mechanism as a whole is distinct from the components and their behaviors and cannot be reduced to them. The fact that one can *derive* information about one from information about the other may suggest to someone that they are not really distinct. But this way of thinking does not follow from the approach I have offered. Recall the approach I have presented holds that the behavior of the mechanism as a whole is not explained in terms of the behaviors of its components individually. The way to see this is to observe that the features appealed to in the explanation in line 2 concern the components’ behaviors *and their organization*. It is not merely the components that do the work in the explanation. The explanation appeals to how the components have been organized together in such a way that they result in the behavior. This organizational property of the mechanism is not an aspect of the lower-level itself but exists with the higher-level parts-plus-their-organization.^9^ So the explanatory scheme provided is consistent with the earlier point that the mechanism’s behaviors should be understood nonreductively. We do not derive this organizational property from the lower-level itself and so this is not a feature to which the mechanism’s behavior can be reduced.

One may think that an approach to explanation that views them as I have described will have to view mechanisms reductively. But this would be a concern only if one overlooked the role of organization among the components. While it may be true that the components are involved in the behaving mechanism, this is not enough to show that the behavior of the whole is due merely to the behavior of the components. As Bechtel notes in one place, “an understanding of the parts alone is not sufficient to understand why the mechanism behaves as it does …. scientists need to consider how the parts and operations are organized” (Bechtel, 2008, p. 151). What the above explanatory scheme helps to illustrate is that this aspect of the explanation is in addition to the reference to the lower-level components. So it is not an explanation of the mechanism’s behavior just in terms of lower-level features.

A second observation is related to this point and concerns the history of debates over reductionism in this area. It should be evident that the account offered is different from an earlier, influential approach to reductionism presented by Nagel (1961) that has been widely discussed (Silberstein, 2002; Ney, 2022). In his account, the notion of reductionism is characterized in terms of the relations between aspects of different theories. Nagel conceived of theories as collections of statements which include laws, and thought that the right way to understand issues about reductionism was to consider theories from different sciences (psychology vs. neuroscience, say) and how these were related. A reduction occurs only if we can state bridge principles connecting the kind terms in the laws of the two theories to one another. In addition, one has to show how the laws of the reduced theory, T1, can be logically derived from the laws of the reducing theory, T2, together with any appropriate boundary conditions that may be involved. So this form of intertheoretic reduction is characterized in terms of the derivation of one set of laws from those of another. This way of thinking about reductionism is different than the account I have presented. While it is true that the derivation of information is important to the explanation of a mechanism’s behavior on my account, this is not a matter of an derivation between theories or laws. Moreover, Nagel’s concern with theories as being the relevant phenomenon is not how I have characterized the notion of reductionism. The account I have offered is not concerned with theories but views things differently.

In the history of debates over reductionism many people have, in fact, moved past approaches focused on relations between theories because there are problems with this sort of approach. For instance, one of the concerns with this approach that was noted is that, if we construe reductionism as requiring correlations between kind terms from different theories (which is a common way the account has been interpreted that I will follow^10^), this would appear too weak to underwrite a genuine form of reduction (Sklar, 1967; Kim, 1998). Knowing that term K1 from T1 can be correlated with term K2 from T2 merely establishes a biconditional relationship between the terms, which is consistent with views that are nonreductionist. This is because even a dualist who accepts the presence of correlations between mental and neurological state kinds can satisfy such an account, though such a person would not be considered a reductionist. To make a claim of reductionism work it seems what is needed is something stronger than a requirement of mere correlations. It was this sort of concern that helped people to see that reductionism should not be conceived as a relationship between theories, but is better characterized in terms of relations between entities that exist in the world. The account I have offered is consistent with this more recent way of thinking about reductionism.

The account offered is concerned with how entities are related to each other. But this should not be taken to mean that there is not a role for theories to play in understanding how entities are structured in the world. The account allows that we can still accept that we need theories at different levels, corresponding to the different levels of entities. To understand this point remember that the account of mechanisms offered holds that there are higher-level behaviors of mechanisms as a whole, and the lower-level components and their behaviors that make them up. The fact that there are different levels of entities helps to explain why there are theories that have been developed in different areas of the sciences. When we are trying to understand why a mechanism behaves as it does, we will sometimes be concerned with the lower-level constituents that contribute to its operation. Understanding the components and their behaviors helps us to understand why the overall behavior of the mechanism occurs; or it may sometimes be that we are just interested in understanding how the components operate in themselves or in relation to others. But knowing about the components does not prevent us from having to study the mechanism at higher levels of organization. The behavior of the mechanism as a whole needs to be studied in its own terms^11^ and in relation to other mechanisms in the environment it interacts with, and with respect to whatever principles are at work at higher levels. These features of the mechanism are not something that can be understood merely by looking to the lower-level components and their behaviors. As a result there is a need for different theories to be offered at different levels because this will help us to make sense of the different aspects that exist.

Finally, it should be noted that this picture of how theories are understood might be further developed to explain what’s useful about having such theories. I have suggested that part of the explanation for this has to do with theories that might be developed at higher levels, which we need to know for an understanding of the various aspects of the mechanisms. These theories might concern how the mechanisms are causally related to other mechanisms in the environment, or they might concern ways of picking out the mechanisms that are of interest, or something else. I think there is more that one would need to say to explain just what these theories are concerned with and how they are able to be useful in the sciences. I would suggest that we can recognize this point without worrying that we need to have all of this worked out at this point to make sense of the account. Given that there are mechanisms in the world with entities and behaviors that exist at different levels, there will be a need for researchers to develop different theories to describe them adequately. The account I have presented can be developed to fit with this point about the features of mechanisms and there is no reason to think the details will change this fact. Regardless of such issues, there will be a need for theories at different levels because of the structure the world exhibits.

## 7. Conclusion

Issues about how mechanistic explanations and reductionism are related have raised a lot of concerns. In this paper, I have tried to offer an account of mechanisms as systems constituted by parts that make them up and say something about how mechanisms so understood can be explained. Once these views have been presented, it helps us to clarify some of the relationships at work in talking about reductionism and mechanisms. The account I have presented suggests that the proper way to understand the mechanisms I have been concerned with is nonreductively. A mechanism should be understood to have behaviors that exist which cannot be reduced to the behaviors of the parts. A behavior of the mechanism is based on the behaviors of the parts present but goes beyond them. The explanation of a mechanism’s behavior has also to include reference to the organizational properties of the mechanism. We can accept that mechanistic explanations refer to components without thinking that is all there is to the explanation.

It is hoped that this way of thinking about these issues provides us with some clarification of mechanisms. Needless to say, I have not attempted to say everything that has to be said about how to understand mechanisms or how they should be explained. Both of these are topics about which more could certainly be said. For example, one issue I noted I have not examined concerns the way one should understand the notion of “levels” used and how this notion can be made more precise. There are different ways of thinking about this notion and it may be useful to consider this more carefully at some point. There are also questions I have not considered about the notion of explanation and how it connects to other issues like the “pragmatics” of explanation (is explanation a contrastive notion, say?), among others. Rather than consider these issues, what I have tried to do is to present a way of thinking about mechanistic explanations and reductionism that offers a way of helping us understand their relationship. It is thought that improving our understanding of their relationship will be useful for addressing these other sorts of issues in the area.

## Author contributions

The author confirms being the sole contributor of this work and has approved it for publication.

## Conflict of interest

The author declares that the research was conducted in the absence of any commercial or financial relationships that could be construed as a potential conflict of interest.

## Publisher’s note

All claims expressed in this article are solely those of the authors and do not necessarily represent those of their affiliated organizations, or those of the publisher, the editors and the reviewers. Any product that may be evaluated in this article, or claim that may be made by its manufacturer, is not guaranteed or endorsed by the publisher.

## Footnotes

^1^A related clarification to make is that in describing mechanisms I will talk of entities and properties, rather than (as some prefer) entities and activities. I think that activity language can be cashed out in terms of the manifestation of capacities when understood properly (*cf.* Psillos, 2004, p. 311), though I do not think this issue will be important in what follows. For discussion of this topic one can see Kaiser (2018).

^2^I say "form of" because there are various part-whole relations that exist and the only one I am concerned with is a part-whole relation involving mechanisms of the type I’ve described.

^3^I will not stop to consider the notion of levels being used (which is a local notion only in contrast to more global ones) because there is recently a large debate about this notion, and considering this would take me too far afield. What I mean to invoke is the notion of levels used by Craver according to which "X’s ϕ-ing is at a lower mechanistic level than S’s ψ-ing if and only if X’s ϕ-ing is a component in the mechanism for S’s ψ-ing" (Craver, 2007, p. 189). It’s possible the notion of levels could be reframed and the arguments of this paper would still go through, as long as there is some appropriate notion of mereological relationships that applies to mechanisms and components existing in a hierarchy. For discussion of different notions of levels one can see Craver (2007) and Potochnik (2017).

^4^In describing his view Craver says, "lower-level components are made up into higher-level [mechanisms] by organizing them spatially, temporally, and actively into something greater than a mere sum of the parts" (Craver, 2007, p. 189).

^5^Another notion holds that a reduction occurs if a mechanism’s behavior is explained merely in relation to its components and their behaviors (Bechtel, 2008, p. 151). This is a rather weak notion which I do not oppose at some level.

^6^Note that others have suggested that we can have an argument-based approach to explanation in a way that differs from Hempel’s account. For example, beyond Hausman, both Kitcher (1981) and Strevens (2008) describe the notion of explanation in terms of arguments, but in ways that depart from Hempel.

^7^It should be observed that the expression "components and behaviors in organization S" in lines 2 and 3 is intended as a summary of whatever components and behaviors and their order exist in the (actual) mechanism in question. These could be listed out with more detail if preferred, although it would make the explanation more complex in certain ways that I would like to avoid here.

^8^I’m thinking of such concerns as the problem of symmetry, the problem of relevance, etc. Another concern with the account is about other forms of representation than sentences. For instance, sometimes researchers describe mechanisms in terms of diagrams. One response is to say that, if the diagrams reveal the relevant components and their behaviors of a mechanism (Bechtel and Abrahamsen, 2005, p. 425), then this information should be translatable into the form of explanation that was described (for an alternative view see Burnston (2016)). But I will not try to develop this point further.

^9^*Cf.* Craver’s claim that "lower-level components are organized together to form higher-level components" (Craver, 2007, p. 189).

^10^There has been some disagreement about how to describe the bridge principles at issue. Richardson (1979) argues that Nagel only required one-way conditionals in his account. But many people have thought that the correlations would have to be at least as strong as bi-conditionals to work. I will follow this approach, though I do not think it affects anything that follows.

^11^*Cf.* section 4.
